# Enhancing the Catalytic Activity of Thermo-Asparaginase from *Thermococcus sibiricus* by a Double Mesophilic-like Mutation in the Substrate-Binding Region

**DOI:** 10.3390/ijms24119632

**Published:** 2023-06-01

**Authors:** Maria Dumina, Dmitry Zhdanov, Alexander Zhgun, Marina Pokrovskaya, Svetlana Aleksandrova, Alexander Veselovsky, Michael El’darov

**Affiliations:** 1 Federal Research Center “Fundamentals of Biotechnology” of the Russian Academy of Sciences, 117312 Moscow, Russia; zhdanovdd@gmail.com (D.Z.); zzhgun@mail.ru (A.Z.); 2 Institute of Biomedical Chemistry, 119121 Moscow, Russiaveselov@ibmh.msk.su (A.V.)

**Keywords:** extremophilic enzyme, hyperthermophile, L-asparaginase, substrate-binding, mutagenesis, enzymatic activity

## Abstract

L-asparaginases (L-ASNases) of microbial origin are the mainstay of blood cancer treatment. Numerous attempts have been performed for genetic improvement of the main properties of these enzymes. The substrate-binding Ser residue is highly conserved in L-ASNases regardless of their origin or type. However, the residues adjacent to the substrate-binding Ser differ between mesophilic and thermophilic L-ASNases. Based on our suggestion that the triad, including substrate-binding Ser, either GSQ for meso-ASNase or DST for thermo-ASNase, is tuned for efficient substrate binding, we constructed a double mutant of thermophilic L-ASNase from *Thermococcus sibiricus* (TsA) with a mesophilic-like GSQ combination. In this study, the conjoint substitution of two residues adjacent to the substrate-binding Ser55 resulted in a significant increase in the activity of the double mutant, reaching 240% of the wild-type enzyme activity at the optimum temperature of 90 °C. The mesophilic-like GSQ combination in the rigid structure of the thermophilic L-ASNase appears to be more efficient in balancing substrate binding and conformational flexibility of the enzyme. Along with increased activity, the TsA D54G/T56Q double mutant exhibited enhanced cytotoxic activity against cancer cell lines with IC_90_ values from 2.8- to 7.4-fold lower than that of the wild-type enzyme.

## 1. Introduction

Among currently used enzymes, L-asparaginase (L-ASNase) remains one of the most important used in medicine, biosensors, and food industry. L-ASNase (EC 3.5.1.1; L-asparagine amidohydrolase) catalyzes the hydrolysis of L-asparagine (L-Asn) to L-aspartic acid and ammonia [1]. It is the first enzyme with antitumor activity to be used in clinical practice after its approval in 1978 [2,3]. Currently, L-ASNase continues to be the mainstay for the treatment of pediatric acute lymphoblastic leukemia and is also used to treat other related blood cancers worldwide [4,5].

L-ASNase therapy is based on L-Asn starvation of susceptible tumor cells that are unable to synthesize their own L-Asn. By hydrolyzing extracellular L-Asn, L-ASNase leads to the death of lymphoblastic cells by apoptosis [6,7].

Relatively recently, the biotechnological area of L-ASNase application has expanded. In 2002, it was discovered that high contents of acrylamide are formed between reducing sugars and L-asparagine during high-temperature (over 120 °C) processing of starchy foods under low humidity in a non-enzymatic Maillard reaction [8].

By hydrolyzing L-Asn, L-ASNase prevents the formation of carcinogenic acrylamide in the Maillard reaction [9]. As aspartic acid cannot participate in the reaction, L-ASNase treatment helps to reduce the formation of acrylamide in commercially fried foods. The method is safe and effective: L-ASNase treatment leads to a decrease in the content of acrylamide up to 99%, and further heating deactivates the enzyme without affecting the properties of the final product [10,11].

Another field of the enzyme application is the development of biosensors. Biosensor systems based on L-ASNase allow to detect the level of L-Asn in medicine and in the food industry [12,13].

Thus, L-ASNase is an enzyme that is widely used in biotechnology under various operating conditions, in particular, in a wide range of temperatures. Most L-ASNases exhibit optimal activity at or near mesophilic temperatures (approximately 30–40 °C) and under mild operating conditions [2]. Obviously, it is difficult for enzymes of mesophilic origin to cover such a wide range of conditions required for their successful application in biotechnology, especially in the food industry. Extremophiles, in particular, thermophiles, which have been reported to produce L-ASNases with unique properties, can expand the number of biotechnologically available L-ASNases [9,14,15,16,17]. According to experimental data, thermophilic L-ASNases (thermo-ASNases) can not only occupy a vacant niche of high-temperature food technologies but also compete with mesophilic enzymes in biomedicine [9,15,16,17].

Due to their superior performance, investigation of thermo-ASNases is of particular interest. Elucidation of the molecular mechanisms that determine the relationship between the activity, stability, and flexibility of thermo-ASNases is critical for both fundamental and applied research in view of their biotechnological significance.

Previously, we have characterized a new promising hyperthermophilic L-ASNase from the archaea *Thermococcus sibiricus* (TsA) [16]. The enzyme is optimally active at 90 °C, stable, and exhibits high specific activity and strong cytotoxic activity toward cancer cells.

Various protein engineering approaches are used in an attempt to improve the properties of known L-ASNases. Based on the traditional directed evolution method, Kotzia and Labrou engineered mutants of mesophilic L-ASNases from *Erwinia carotovora* and *Erwinia chrysanthemi* [18]. A thermostable mutant Asp133Val was obtained from a library of enzyme variants. This powerful approach is highly dependent on the quality and quantity of the generated libraries, and a target biobetter form is hard to obtain [19,20]. Enzyme computational engineering approaches can reduce search screening. Offman et al. successfully constructed a proteolysis resistant mutant of *Escherichia coli* L-ASNase with improved activity by adapting a genetic algorithm of protein modeling in combination with molecular dynamics flexibility studies [21]. The sequence-based approach is a useful technique for engineering desired forms of proteins, in particular with unsolved structures, based on the analysis of well-characterized homologous enzymes. By employing multiple sequences alignment in tandem with homologous modeling, mutant form of *Bacillus subtilis* L-ASNase with improved thermostability was obtained [22]. Based on sequence alignment and structure superposition of thermophilic and mesophilic L-ASNases, Li et al. identified two residues that affect their thermostability [23].

Here, we report an attempt to improve TsA properties by the conjoint substitution of two so-called “special residues”. Based on available experimental data, amino acid residues that are highly conserved among thermo- but not mesophilic L-ASNases (“special residues”) can affect the thermoactivity and thermostability of thermo-ASNases [23].

The single mutations had been previously found to decrease L-asparaginase activity of wild-type thermo-ASNases [23] or were performed on a low-activity enzyme [15]. In this study, the TsA double mutant displayed ~240% relative L-asparaginase activity compared to the wild-type enzyme.

## 2. Results

### 2.1. Prediction of Mutations for Improving Thermo-Asparaginase Activiry

A pairwise sequence comparison of thermophilic/mesophilic enzymes is an effective way to explore key amino acid substitutions affecting the activity and stability of enzymes evolutionarily adapted to different working temperatures.

**Figure 1:**
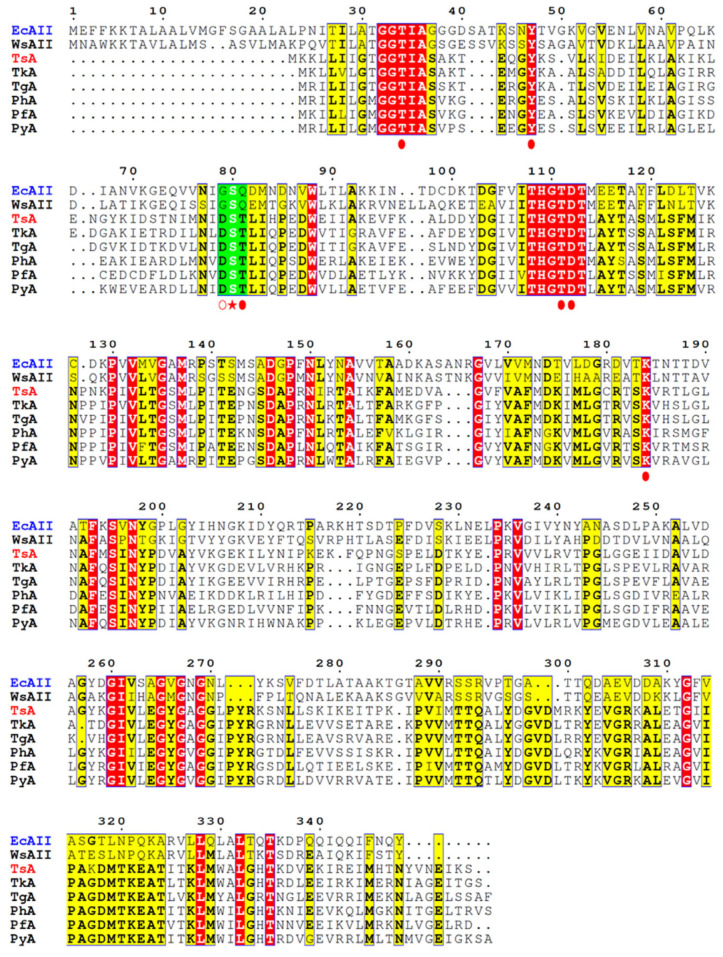
Amino acid sequence comparison of mesophilic L-asparaginases (L-ASNases) from *Escherichia coli* (EcAII, type II—AAA23445.1, marked in blue) and *Wolinella_succinogenes* (WsA, type II—WP_011138590.1) and thermophilic L-ASNases from *Thermococcus sibiricus* (TsA, WP_015849943, marked in red), *Thermococcus kodakarensis (*TkA, WP_011250607.1), *Thermococcus gammatolerans* (TgA, WP_015859055), *Pyrococcus horikoshii* (PhA, WP_010884185), *Pyrococcus furiosus* (PfA, WP_011013191), *Pyrococcus yayanosii* (PyA, WP_013906452). Identical amino acid residues are marked in red, conserved residues are shown in yellow, and strongly conserved residues crucial for the catalytic activity of selected L-ASNases are indicated by red circles. The substrate-binding Ser residue is marked by a red star, and residues adjacent to the substrate-binding Ser are marked by empty or filled red circles, these residues are boxed in green. ESPript 3.0 was used for multiple sequence alignments [28].

Studies of *E. coli* L-ASNase II (EcAII), used as a representative L-ASNase of mesophilic origin, revealed that in the specific reaction, Thr12, Ser58, and Thr89 of processed form (native signal peptide cleaved) “clamp” and stabilize the substrate [24,25,26,27]. Ser58 is directly involved in substrate binding in EcAII, and its substitution decreases L-ASNase activity [25]. Gly57 and Gln59 are located on either side of the substrate-binding Ser58 residue (Figure 1). Gly57 is reported to play a key role in L-asparaginase-specific activity [23], while Gln59 assists substrate binding and modulates substrate specificity [26,27].

According to the alignment of amino acid sequences, TsA contains residues that are highly conserved among thermo-ASNases. Based on crystallographic studies of thermo-ASNases, the residuesThr12, Tyr22, Ser55, Thr56, Thr86, Asp87, and Lys157 of TsA are crucial for the catalytic activity of the enzyme (Figure 1) [29,30,31]. Of particular interest is the role of “special” residues adjacent to the substrate-binding Ser55. The catalytic Ser55 in TsA is surrounded by Asp54 and Thr56 residues, which are highly conserved in thermo-ASNases but not in L-ASNases of mesophilic origin (Figure 1, Supplementary Materials Figure S1). Asp54 and Thr56 of TsA are substituted in the *E. coli* enzyme by Gly 57 and Gln59, respectively (Figure 1).

The suggestion is that the triad—either mesophilic G**S**Q or thermophilic D**S**T—is tuned to efficiently bind the substrate. To improve thermo-ASNase TsA activity, the mesophilic-like G**S**Q combination may be more effective in balancing substrate binding and conformational flexibility.

The effect of the two simultaneous mesophilic-like substitutions of residues adjacent to the substrate-binding Ser in the highly conserved D**S**T triad of thermo-ASNases (Figure 1) has not been studied. Nevertheless, single mutations—Asp (thermo-ASNase) → Gly (meso-ASnase EcAII) and Thr (thermo-ASNase) → Gln (meso-ASNase EcAII)—have been previously tested with varying degrees of success.

Previously, in an attempt to improve the enzymatic properties of a low-activity hyperthermophilic enzyme from *Pyrococcus furiosus* (PfA), Thr53 corresponding to Thr56 of TsA was replaced by Gln found in EcAII at the same position. It was reported that the T53Q substitution affected PfA substrate affinity, but the effect of the mutation on PfA activity was not provided [15].

**Figure 2:**
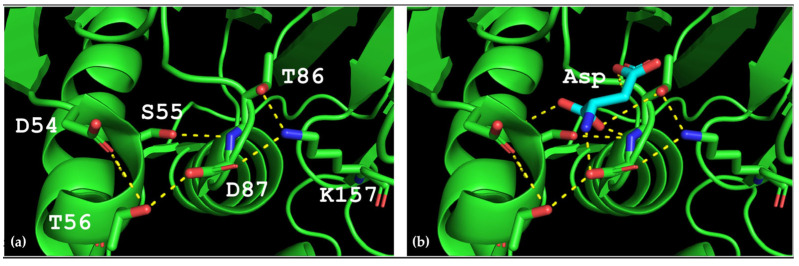
Predicted structure of the active site of L-ASNase from *T. sibiricus*. (**a**) Hydrogen bond network in the TsA active site; (**b**)—model of aspartate (product) position in the TsA active site. Yellow dash lines—H-bonds.

The spatial structure of L-ASNase from *T. sibiricus* was absent, so the model of this enzyme was designed using method of homology modeling using the crystal structure of thermo-ASNase from *Thermococcus kodakarensis* (TkA) as a template. Thr56 of TsA is located at the bottom of the active site pocket and is involved in multiple interactions (Figure 2a). The residues Asp54, Ser55, Thr56, Thr86, Asp87, and Lys157 are involved in the main interactions of this part of the active site. They form a network of H-bonds that can restrict loop (residues 85–88) movement. A model of the TsA-substrate complex was done by incorporating aspartate from the *E. coli* asparaginase complex (pdb 3eca) into the model after spatial alignment of L-ASNase and the TsA model. It was shown that Thr86, Asp54, and Lys157 are also involved in the interaction with the substrate (product) (Figure 2b).

Analysis of the TsA model showed that the replacement of Thr56 with the corresponding Gln residue of EcAII can disrupt the H-bond network by shifting the position of the Gln amide group away from Asp87, but toward Asp54. Destruction of the H-bond network will increase the flexibility of the loop and improve the enzymatic properties of the thermo-ASNase. On the other hand, due to small distances, the appearance of bulky Gln instead of Thr in TsA can cause the Gln and Asp residues to move away from the substrate, resulting in a decreased efficiency of formation of the substrate–enzyme complex. To prevent this, it is preferable to reduce the size of the near located residues. Thus, if Thr is replaced with Gln, a simultaneous mesophilic-like substitution is needed to provide high specific activity of thermo-ASNase—D**S**T → G**S**Q.

Additionally, it was previously reported that the single D → G mutation reduces L-asparaginase activity of all thermo-ASNases studied due to impaired substrate holding by the catalytic Ser residue and adjacent Gly after Asp replacement [23].

Based on previous experimental data and homology modeling, the conjoint substitution in the highly conserved for thermo-ASNases D**S**T triad with the resulting G**S**Q combination may be beneficial in increasing the catalytic activity of TsA.

### 2.2. Site-Directed Mutagenesis, Expression and Recombinant Enzyme Purification

In an attempt to study the role of the conjoint substitution adjacent to the substrate-binding Ser, a TsA double mutant was developed. The mutant was constructed on the basis of the codon-optimized gene *tsA_mod*, which was previously developed for more efficient heterologous expression of the archaeal protein in *E. coli* cells [16]. Site-directed mutagenesis of *tsA_mod* inserted into the pET-28a (+) vector was performed. The constructed plasmid was transformed into the host *E. coli* BL21 (DE3) for the expression of the D54G/T56Q mutant. The wild-type and mutant enzymes were purified by ion-exchange chromatography under the same experimental conditions with final yields shown in Table 1.

**Figure 3:**
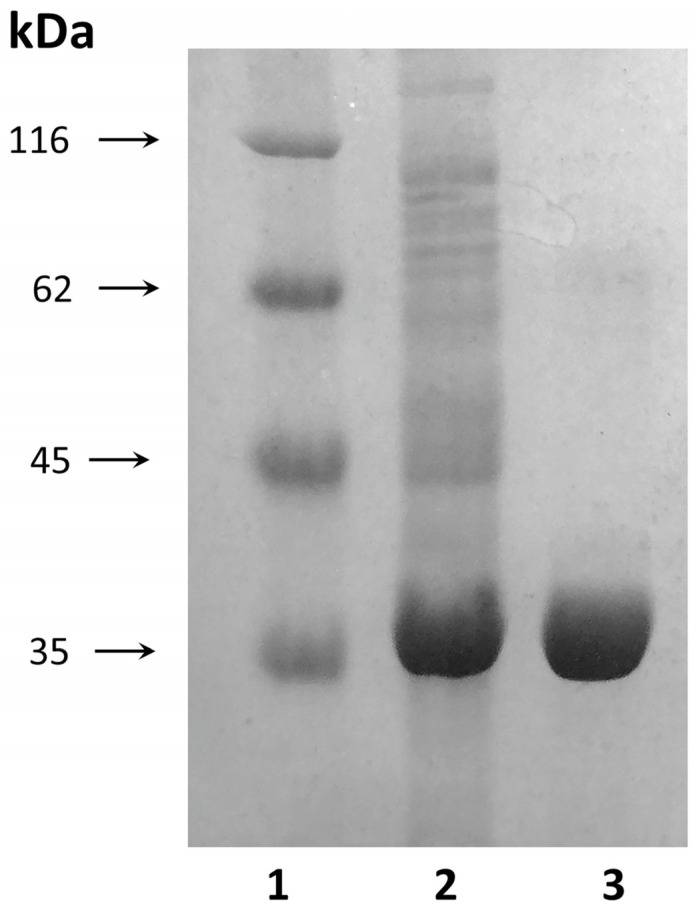
SDS-PAGE analysis: 1—protein molecular weight marker. 2—the D54G/T56Q mutant in cell-free homogenate at a concentration of 20 µg; 3—purified D54G/T56Q at a concentration of 10 µg.

The molecular weight of the purified D54G/T56Q mutant was ~37.5 kDa when analyzed with SDS-PAGE (Figure 3).

### 2.3. Enzymatic and Kinetic Properties of TsA and Its Double Mutant

The enzymatic properties of the double-mutant D54G/T56Q compared with the wild-type enzyme were investigated. According to our previous data, TsA is one of the most active thermo-ASNases, exhibiting high hydrolysis activity toward L-Asn [16]. Analysis of the mutant specific activity revealed that the conjoint substitution D54G/T56Q led to a significant increase in activity toward L-Asn—from 2066.1 U/mg for TsA to 5037.7 U/mg for the double mutant (Table 1).

Substrate specificity experiments demonstrated that the D54G/T56Q mutant has lower glutaminase activity than the wild-type enzyme. The relative L-glutaminase activity was 5% for the mutant and 7% for wild-type TsA.

The kinetic properties of the enzymes were assessed. The K_m_ and V_max_ of the mutant enzyme for substrate L-Asn were found to be 6 mM and 8170 µM/min, respectively. Under the same experimental conditions, the K_m_ and V_max_ of the wild-type thermo-ASNase TsA were estimated to be 3 mM and 4020 µM/min, respectively.

Thus, for the double mutant, the mesophilic-like substitution in the active site caused a simultaneous increase in the specific activity and K_m_ and V_max_ values compared with the wild-type enzyme.

### 2.4. The Dependence of Specific Activity on Temperature and pH

**Figure 4:**
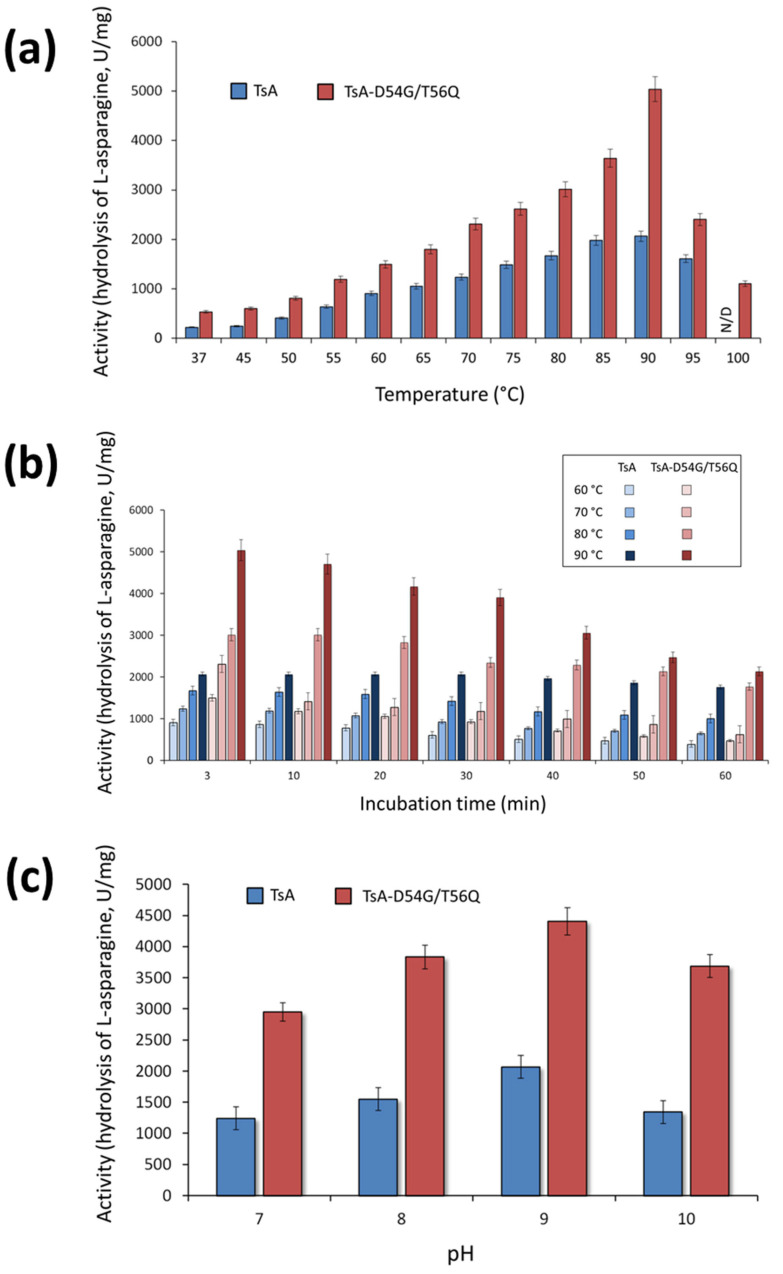
Dependence of (**a**) absolute activity and (**b**) stability of TsA and its double mutant TsA-D54G/T56Q on temperature. The temperature–stability profile is expressed as specific activity after 0–60 min of incubation. (**c**) Effect of pH on the catalytic activity of the enzymes. ND—not determined value.

The activity of the TsA double mutant was evaluated in the temperature range from 37 to 100 °C. The results revealed that mutations D54G/T56Q did not shift the temperature optimum (T_opt_) of the enzyme (Figure 4a). TsA and its double mutant exhibited maximum activity at 90 °C (Figure 4a). Interestingly, the mutant D54G/T56Q displayed 22% relative activity at 100 °C, while no activity was observed for the wild-type enzyme at this temperature (Figure 4a).

The results indicated that TsA-D54G/T56Q has a sharp and relatively narrow temperature optimum (Figure 4a). The optimum curve of the wild-type enzyme has a broader maximum, indicating that it is relatively less sensitive to changes in temperature. The time-dependent loss of enzyme activity at various temperatures was higher for the mutant than for the wild-type enzyme (Figure 4b). Nevertheless, the absolute value of the specific activity was higher for the mutant than for the native TsA in the entire temperature range (Figure 4a,b). At the optimum temperature, the TsA double mutant activity reached 240% of the activity the wild-type enzyme, and both TsA-D54G/T56Q and TsA exhibited high activity, exceeding 1000 U/mg, over a wide temperature range (Figure 4a).

The enzymatic activity of TsA-D54G/T56Q compared to TsA was evaluated in the pH range of 4.0 to 10.0 in different buffer systems. The pH dependence of the activity was quite similar. TsA-D54G/T56Q exhibited maximum activity at pH 9.3. Both enzymes displayed high relative activity in a pH range of 7.0–10.0 independent of the buffer system. The absolute value of the mutant specific activity was 2-fold higher than that of native TsA in a working range of pH 7.0–10.0 (Figure 4c).

### 2.5. Effect of Urea and Metal Ions on Specific Activity of the Mutant and Wild-Type Enzymes

The wild-type thermo-ASNase TsA was stable in the presence of urea. Pre-incubation of TsA with urea until a final concentration of 6 M did not result in a significant decrease in enzyme activity. The double mutant D54G/T56Q was even more stable in the presence of higher urea concentrations, retaining 54.6% of its initial activity when incubated with 8 M urea.

The D54G/T56Q mutant displayed a nearly identical response as TsA when various metal cations Ni^2+^, Cu^2+^, Mg^2+^, Zn^2+^, Ca^2+^, Fe^3+^ and EDTA at a concentration of 10 mmol/L were added to the reaction mixture. No significant change in activity of the enzymes was observed in the presence of metal ions or EDTA, except for Fe^3+^. The addition of Fe^3+^ led to a sharp decrease in L-asparaginase activity by more than 82.5% for the mutant and 85.0% for the wild-type enzyme.

### 2.6. Investigation of the Double-Mutant Cytotoxic Activity

Previously, we have shown that the thermo-ASNase from *T. sibiricus* displays cytotoxic activity toward cancer cell lines, while normal cells are almost insensitive to its activity [16]. To test how the double mutation D54G/T56Q affects the cytotoxic activity of the enzyme, a cytotoxicity test and an apoptosis induction assay were performed.

**Figure 5:**
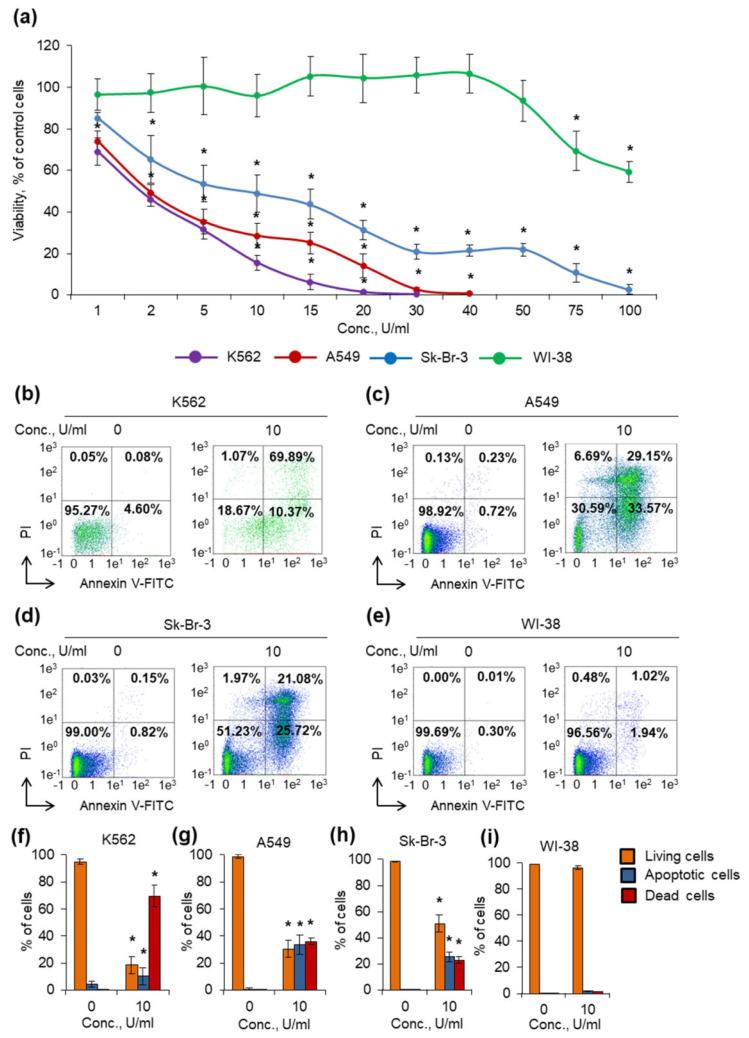
TsA-D54G/T56Q capacity for inducing apoptosis in cancer cells. Cancer cells K562, A549, Sk-Br-3 or normal fibroblasts WI-38 were cultivated for 72 h in the presence of the enzyme. The concentration range was 1–100 U/mL. (**a**) Cell toxicity determined by MTT assay. The cells were incubated with 10 U/mL of the enzyme, followed by labelling with Annexin V-FITC and propidium iodide (PI), and analysis by flow cytometry. (**b**–**e**) Representative examples of flow cytometry diagrams showing live (lower left quadrants), apoptotic (lower right quadrants), and dead (two upper quadrants) cell ratios. (**f**–**i**) Histograms of live, apoptotic, and dead cells as determined by flow cytometry *n* = 4. * *p* ≤ 0.05 compared to untreated control cells.

Human cancer cells and normal fibroblasts were cultivated in the presence of the enzyme added at various concentrations. After 72 h of incubation, cell viability and apoptosis induction were measured (Figure 5).

Among cancer cells, K562 cell line demonstrated the highest sensitivity, and the mutant enzyme was able to reduce cell viability even at the concentration of 1 U/mL (Figure 5a). The Sk-Br-3 cell line demonstrated the highest resistance: more than 20% of cells remained alive at a concentration of 50 U/mL. Among cancer cells, A549 cell line demonstrated moderate sensitivity for the double mutant D54G/T56Q. WI-38 cells (normal human fibroblasts) were almost insensitive to the enzyme. The significant decrease in cell viability was observed at the highest concentrations of 75 and 100 U/mL.

The anticancer activity of the wild-type enzyme TsA and its double mutant D54G/T56Q, assessed by comparing the IC_50_ and IC_90_ values for the tested cell lines, is presented in Table 2.

The common mechanism of L-ASNases action is the induction of apoptosis after L-asparagine deprivation [32,33]. We studied the proportion of apoptotic cells using flow cytometry after cell treatment with 10 U/mL D54G/T56Q and labeling phosphatidyl serine on cell membranes with annexin V-FITC and cell DNA with PI. The results of apoptosis measurement and MTT test were in accordance. The double mutant could induce apoptosis more efficiently in K562 cells and less than 20% of K562 cells remained alive after incubation (Figure 5b,f, Supplementary Materials Figure S2). Sk-Br-3 and A549 cells were more resistant: 51 and 30% of cells remained alive, respectively (Figure 5c,d,g,h). The enzyme did not induce apoptosis in normal fibroblasts WI-38 (Figure 5e,i).

The overall results demonstrated that the double mutant D54G/T56Q enhances the cytotoxic activity of thermo-ASNase against cancer cell lines, whereas normal cells were almost insensitive even to the highest enzyme dose (Table 2). The IC_90_ values were 2.8-fold to 7.4-fold lower for the mutant D54G/T56Q than for the wild-type enzyme.

## 3. Discussion

Mutations usually have a variety of abilities to fine-tune the functions of enzymes [34,35]. No general guidelines to enhance the activity of the promising wild-type enzyme have been established. For thermo-ASNase TsA, the approach based on the simultaneous mesophilic-like substitutions of residues adjacent to the substrate-binding Ser in the highly conserved D**S**T triad resulted in a 2-fold increase in activity.

Homology models revealed that a single mutation—either Asp (thermo-ASNase) → Gly (meso-ASNase EcAII) or Thr (thermo-ASNase) → Gln (meso-ASNase EcAII)—impairs proper substrate binding. Nevertheless, restoring the entire mesophilic-like triad G**S**Q in TsA after double mutation improved its activity.

In EcAII, the downstream residue Gln59 assists the Ser58 residue in substrate binding. In thermo-ASNases, the upstream Asp residue adjacent to the substrate-binding Ser is supposed to play the same role. The puzzle is that, in terms of function, the D**S**T triad is “inverse” in thermo-ASNases compared to mesophilic G**S**Q relative the substrate-binding Ser. Thus, both previously used mutational strategies in this region to replace single Asp or Thr residue with their directly corresponding residues of EcAII are not entirely correct from the point of increasing activity.

In thermo-ASNase PfA, the T53Q mutant is reported to have an increased substrate affinity of 8.3 mM compared to 12.1 mM for the wild-type enzyme. At the same time, the substitution of conservative Thr53 correlates with a more than 2-fold loss in catalytic efficiency [15]. The combination of three adjacent D**S**Q residues involved in substrate binding and stabilization can lead to an increase in docking strength in thermo-ASNases. At high docking strength, the docking lifetime is longer than the time required for catalysis [36].

In turn, in thermo-ASNases *Pyrococcus yayanosii* (PyA) and *Thermococcus gammatolerans* (TgA), the Asp → Gly replacement with the resulting “mixed” triad residues G**S**T impaired substrate binding, increased flexibility around the binding Ser residue due to the deficiency of polar contacts [23]. Decreased binding function of the Ser residue caused dramatic loss of PyA and TgA activity (Table 3).

Although single mesophilic-like substitutions of residues adjacent to the substrate-binding Ser—Thr→Gln and Asp→Gly reduced the catalytic efficiency or activity of thermo-ASNases, the simultaneous introduction of their combination in TsA increased the specific activity of the enzyme (Table 3).

**Figure 6:**
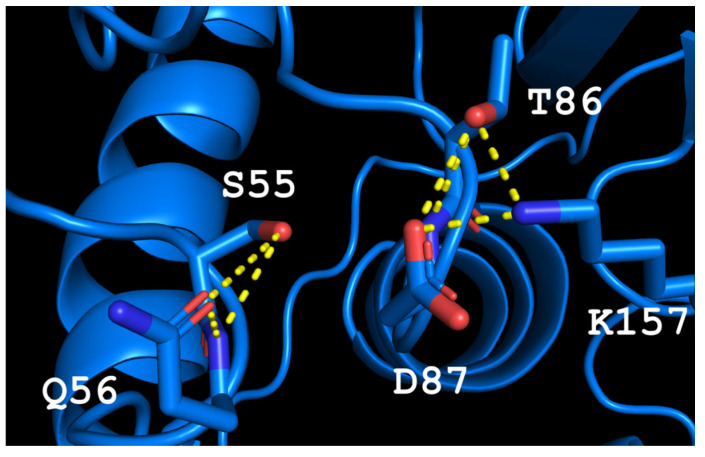
The model of the TsA-T56Q/D54G active site. Yellow dash lines—H-bonds.

The predicted model of TsA showed that the structure of the active site is rigid, stabilized by a network of H-bonds. The T56Q mutation disrupts this network, increasing the flexibility of the active site, but the appearance of a bulky glutamine side chain can affect the interaction of the substrate in the active site. This observation leads to the mutation of the second residue to increase the available volume to accommodate the glutamine side chain. Thus, the second mutation D54G was chosen. The possible conformation of residues in the active site of the double mutant is shown in Figure 6. The resulting G**S**Q triad, fine-tuned to substrate binding in mesophilic EcAII, provided proper “clamping” and stabilizing of the substrate during the catalytic process in TsA thermo-ASNase. The increased flexibility of the structure around the active site may promote reorientation, substrate binding and increase the specific activity.

Analysis of the mutant specific activity revealed that the conjoint substitution D54G/T56Q led to a significant increase in activity toward L-Asn—from 2066.1 U/mg for TsAwt to 5037.7 U/mg for the double mutant. In addition, a slight decrease in glutaminase activity was observed: from 7% for TsAwt to 5% for the mutant D54G/T56Q. Consistent with our data, in studies of EcAII, it was shown that Gly57 and Gln59 residues can modulate the substrate specificity of L-ASNase [23,26,27].

By analyzing the effect of temperature on the activity and stability of the double mutant, it was revealed that there was no shift in the optimum temperature, while the sensitivity to temperature changes and time-dependent loss of absolute enzyme activity increased.

The effect of a single mutation corresponding to D54G of TsA—D51G of the thermo-ASNase PyA and D52G of the thermo-ASNase TgA was studied by Li et al. [23]. The mutated enzymes PyA D51G and TgA D52G displayed a lower optimum temperature: the T_opt_ values were 30 °C and 25 °C lower than those of the wild-type enzymes, respectively (Table 3) [23].

In reverse experiments using representative mesophilic L-ASNases from *E. coli* EcAII and *Bacillus subtilis* BsAII, site-directed mutagenesis was carried out, where residues G57 of EcAII and G107 of BsAII were replaced with the corresponding residue D54 of TsA. In this study, EcAII-G57D showed higher T_opt_ than wild-type EcAII [23]. In contrast, for BsAII-G107D, no shift in temperature optimum was found.

No shift in the optimum temperature was reported after the substitution of Thr53 in PfA corresponding to Thr56 of TsA [15].

Li et al. have shown that the replacement of the corresponding residue of TsA D54G—PyA D51G and TgA D52G—led to a decrease in the thermostability of thermo-ANSases, regardless of their source [23], which is consistent with our experimental data. Conversely, the substitution G→D improved thermostability for both mesophilic L-ASNases—EcAII and BsAII [22,23].

The overall results confirm that the amino acid residue in the corresponding position—D54 of TsA, D52 of TgA, D51 of PyA, G57 of EcAII, and G107 of BsAII—is one of the key residues responsible for the thermostability of L-ASNases, but this residue does not necessarily affect L-ASNase optimum temperature.

Analysis of cytotoxic activity in vitro revealed that the double mutant D54G/T56Q was more active against cancer cell lines than TsAwt. The IC_90_ values were 2.8-fold to 7.4-fold lower for the mutant than for the wild-type enzyme. A drastic increase in cytotoxic activity was also previously reported for the PfA mutant T53Q [15].

The triad of L-ASNases studied in this work is actually a special case illustrating a key substitution between thermophilic and mesophilic enzymes. Known thermo-ASNases, including TsA, avoid the uncharged polar residue Gln [14]. Indeed, EcAII contains 13 Gln residues, while only 3 glutamines are present in TsA. In general, a decreased content of Gln residues in enzymes of thermophilic origin is a common feature. At higher temperatures, the frequency of spontaneous chemical modifications, such as deamidation, increases multiple folds [38]. The reaction rate of deamidation increases 350-fold at 100 °C compared to 37 °C. The deamidation mechanism is known for two residues—Asn and Gln [39]. The absence of a unique unstable Gln residue in thermo-ASNases, which promotes substrate binding in mesophilic GSQ triad, is supposed to minimize the possibility of chemical modifications and prevent blocking of protein functioning.

A small Thr residue of thermo-ASNases involved in multiple interactions in this region contributes to protein packing density and structural rigidity at high temperatures.

If the environment is cooler, resistance to spontaneous modification and thermal stability are not so important. The fitness of mesophiles is determined by enzyme activity, which is affected by the flexibility of the protein structure. According to structure modeling, the Gln residue in the mesohilic EcAII G**S**Q triad is preferable for increasing conformational flexibility of L-ASNase active site.

Taking into consideration, that susceptible to destruction and modification at high temperatures amino acid residues can also present in thermophilic and even hyperthermophilic enzymes if they are involved in specific stabilizing interactions or bring a special function and/or are inaccessible to the solvent [39], the T56Q mutation was performed. The thermolabile Gln residue was introduced relying on conformational environment and mobile lid cover the active site as protection factors. Neighboring Asp was replaced by Gly to keep the proper active site architecture favoring catalysis.

In this study, the conjoint substitution D54G/T56Q increased flexibility around the active site of TsA, facilitating conformational changes upon substrate binding and resulting in increased activity. As expected, the mutant displayed slightly lower heat stability. Enhanced rigidity of thermophilic enzymes correlates with increased thermal stability and vice versa [39,40].

An approach based on pairwise sequence comparison of thermophilic/mesophilic L-ASNases and substitution of highly conserved amino acid “special residues” by beneficial non-conflicting residues of mesophilic EcAII improved the catalytic activity of TsA thermo-ASNase. In TsA, the G**S**Q triad, fine-tuned for substrate binding in mesophilic EcAII, increased activity more than 2-fold at 37 °C and 90 °C. D54G/T56Q double mutant with increased activity at 90 °C can be efficiently used in the high-temperature food industry. The increased activity at 37 °C and cytotoxicity of TsA-D54G/T56Q make it possible to compete with other L-ASNases in biomedicine.

## 4. Materials and Methods

### 4.1. Reagents, Enzymes, and Strains

All chemicals used in the experiments were of analytical grade and purchased from Fluka Chemical Corp. (Fluka Chemie GmbH, Buchs, Switzerland), Serva (SERVA Electrophoresis GmbH, Heidelberg, Germany), Paneco (Moscow, Russia) or Reachem (Moscow, Russia). The plasmid pET28a-TsA harboring gene *tsA_mod* (GenBank accession No. MW981255) was previously obtained in our laboratory and used in the study [16]. The enzymes used to construct the plasmid harboring the mutant form of *tsA* were purchased from SibEnzyme (SibEnzyme-M, Moscow, Russia). The expression strain *E. coli* BL21 (DE3) was purchased from Novagen (Madison, WI, USA).

### 4.2. Site-Directed Mutagenesis

Gene *tsA_mod* optimized for expression of archaeal L-ASNase TsA in *E. coli* cells and inserted into the pET-28a(+) vector was used as a template for PCR-based site-directed mutagenesis [16]. The oligonucleotide sequences used to construct the site-directed mutant were 5′-CAACATCATGAATATCGGTAGCCAGCTGATTCATCCGGAAG and 5′-CTTCCGGATGAATCAGCTGGCTACCGATATTCATGATGTTG.

DpnI-treated PCR products were transformed into competent *E. coli* cells for expression. DNA sequencing was used to verify the mutations.

### 4.3. Expression and Purification of the TsA Double Mutant D54G/T56Q

Expression and purification of the recombinant enzymes were performed as previously described with minor modifications [16]. Recombinant strains were grown in the media containing kanamycin (0.05 mg/mL). Protein expression was induced by adding 0.2% lactose. After cultivation for an additional 17–20 h, the cells were pelleted by centrifugation at 4000× *g* for 15 min. Eight grams of cooled biomass was suspended in 100 mL of buffer (20 mM sodium phosphate buffer pH 7.2, 1 mM glycine, 1 mM EDTA), destroyed by ultrasound, and purified as previously described [16].

Protein concentration was determined using the method of Sedmak [41]. SDS-PAGE was performed to test protein purity [42].

### 4.4. Determination of Enzyme Activity and Kinetic Parameters: Evaluation the Effect of Temperature and pH

The activity of L-ASNases was determined using direct Nesslerization [43,44]. The reactions were performed at 90 °C in Tris–HCl buffer (0.05 M, pH 9.0) for TsA and in glycine–NaOH buffer (0.01 M, pH 9.3) for TsA double mutant. Specific enzyme activity was expressed in U/mg protein.

Determination of D54G/T56Q kinetic parameters was performed in glycine–NaOH buffer (0.01 M, pH 9.3) containing 20–250 μM L-asparagine at 90 °C [45].

Activity of D54G/T56Q was analyzed in the temperature range from 45 to 100 °C with 5 °C increments. Additionally, activity was measured at a physiological temperature of 37 °C. The mixture was assayed in glycine–NaOH buffer (0.01 M, pH 9.3).

Thermostability of TsA-D54G/T56Q was assayed by detecting the residual activity of the enzyme that had been preincubated at temperatures ranging from 60 to 90 °C with 10 °C increments at pH 9.3.

The pH dependence of the mutant activity was analyzed by measuring the enzyme activity at different pH values at 90 °C in four buffer systems: sodium acetate (0.05 M, pH 4.0–6.0), sodium phosphate (0.05 M, pH 6.0–7.0), Tris–HCl buffer (0.05 M, pH 7.0–9.0), and glycine–NaOH buffer (0.05 M, pH 9.0–10.0).

For the wild-type enzyme TsA, experiments were performed as previously described [16].

### 4.5. Chemical Denaturation Studies and Effect of Various Metal Ions

Stability assay in the presence of 0–8.0 M urea was performed in glycine–NaOH buffer (0.05 M, pH 9.3) for TsA-D54G/T56Q or Tris–HCl buffer (0.05 M, pH 9.0) for TsA as previously described [16]. The measured activities were compared with the activity of the enzymes without urea addition at 90 °C.

To evaluate the effects of metal ions on enzyme activity, various cations (Ni^2+^, Cu^2+^, Mg^2+^, Zn^2+^, Ca^2+^, Fe^3+^) and EDTA were added at a concentration of 10 mM. The enzyme activity was assayed at 90 °C and optimum pH values by adding L-asparagine and the corresponding metal ion(s) or EDTA. The measured activities were compared with the activity of the enzymes without metal ion or EDTA addition under the same conditions.

### 4.6. Determination of Cytotoxic Activity

The following cells (all from ATCC, Manassas, VA, USA) were used in the study. Human mammary gland adenocarcinoma Sk-Br-3, lung epithelial carcinoma A549, and chronic myelogenous leukemia K562 cell lines were used as cancer cells, whereas WI-38 normal human fibroblast were used as control non-cancer cells. Sk-Br-3, A549, and K562 cells were grown in RPMI-1640 medium, and WI-38 cells were grown in DMEM. Growth media were purchased from Gibco (Thermo Fisher Scientific Inc., Waltham, MA, USA). All media were supplemented with 5% fetal bovine serum and 1% sodium pyruvate (Thermo Fisher Scientific Inc., Waltham, MA, USA). The cells were grown at 5% CO2/95% air at 37 °C. Cells were tested for mycoplasma contamination before each experiment using the Mycoplasma Detection Kit PlasmoTest (InvivoGen, San Diego, CA, USA).

Effect of the enzyme on cell growth and proliferation was examined via an MTT assay [46] by measuring cell viability. The cells were trypsinized with Trypsin-EDTA 0.25% (Thermo Fisher Scientific Inc., Waltham, MA, USA), seeded in 96-well plates (TPP, Trasadingen, Switzerland) at a concentration of 1 × 10^4^ cells per well, and incubated for 24 h. The enzyme was added to culture medium in the range of concentrations 1–100 U/mL and cells were incubated for 72 h. After that the solution of 3-(4,5-dimethyl-thiazol-2-yl)-2,5-diphenyltetrazolium bromine (MTT reagent, Serva, Heidelberg, Germany) at the concentration 5 mg/mL was added, and cells were incubated for 4 h more, followed by cell lysis with DMSO (Helicon, Moscow, Russia) and absorbance measurement at 570 nm. Non-treated cells were used as control. The viability of treated cells was expressed as a percentage relative to control. IC_50_ and IC_90_ values (the concentration of the enzyme where the response is reduced by 50% and 90%, respectively) were calculated from curve-fitting equations.

To measure apoptosis, cells were incubated with 10 U/mL of the enzyme for 72 h in T-25 flask. Incubated cells were resuspended in PBS and labeled with annexin V-FITC and propidium iodide (PI) from a FITC Annexin V/Dead Cell Apoptosis kit (Life Technologies, Carlsbad, CA, USA) according to the standard manufecturer’s protocol. Flow cytometry was performed using a MACS Quant Analyzer 10 (Miltenyi Biotec GmbH, Bergisch Gladbach, Germany) to count 5 × 10^4^ cells at each time point [47].

### 4.7. Statistical Analysis

The data from three parallel experiments were presented as the mean value ± standard error of mean. One-way analysis of variance (ANOVA) using Microsoft Excel (version 2016) was used for statistical analysis.

In the measurement of cell viability and apoptosis induction, Student’s *t*-test statistical analysis was used by Statistica software (version 9.0, StatSoft, Tulsa, OK, USA). Differences *p* ≤ 0.05 were considered significant. The results are presented as the mean ± standard error of the mean (SEM).

### 4.8. Structure Modeling

The spatial structure of L-ASNase from *T. sibiricus* was designed using SWISS-MODEL [48] (https://swissmodel.expasy.org/, accessed on 10 April 2023). The structure of L-ASNase from *T. kodakarensis* was selected (PDB ID: 5ot0) as template, with 62.69% identity to TsA. The mutations were done using PyMol program. Rotamers were selected that did not overlap neighboring residues followed by structure optimization by minimization of energy. Tripos force field was used. Partial atomic charges were calculated using Gasteiger-Huckel method.

## Figures and Tables

**Table 1:** Purification of the wild-type and mutant enzymes.

TsA	Purification Step	Total Protein, mg	Total Activity, U	Specific Activity, U/mg	Yield, %	Purification Fold
Wild-type	crude enzyme	165.0	108,124.5	655.3	100.0	-
	purified enzyme	45.0	92,974.5	2066.1	86.0	3.2
D54G/T56Q	crude enzyme	632.7	1187,704.4	1877.2	100.0	-
	purified enzyme	189.0	952,125.3	5037.7	80.2	2.7

The purification factor was calculated on the basis of specific activity of the purified enzyme and the enzyme in the crude extract sample.

**Table 2:** Values of IC_50_ and IC_90_ of mutant enzyme and wild-type TsA for treated cells.

Cell Line	IC_50_, U/mL		IC_90_, U/mL	
	TsA [16]	TsA-D54G/T56Q	TsA [16]	TsA-D54G/T56Q
K562	1.5	1.7	13.6	4.9
A549	6.6	2.0	45.4	6.5
Sk-Br-3	15.8	3.3	82.1	11.1
WI-38	97.1	140.2 *	>300	>530 *

Mean from eight MTT assays are shown. The estimated errors were in the range of ±5% of the mean. *—Theoretically calculated. Not confirmed experimentally.

**Table 3:** Effect of residues adjacent to the substrate-binding Ser on the main characteristics of thermophilic L-ASNases.

Combination of Residues Adjacent to the Catalytic Active Ser *	Source of L-ASNase	Abbreviation	Topt	Km Value, mM	Specific Activity, U/mg	References
mesophilic G**S**_58_Q	*E. coli*	EcAIIwt	37	0.018	235	[23]
D**S**_52_T	*P. furiosus*	PfAwt	80–85	12.1	550	[15,37]
D**S**_52_Q	*P. furiosus*	PfAmut	80	8.3	ND	[15]
D**S**_52_T	*P. yayanosii*	PyAwt	95	6.5	1483	[23]
G**S**_52_T	*P. yayanosii*	PyAmut	65	3.1	341	[23]
D**S**_53_T	*T. gammatolerans*	TgAwt	85	8.3	5381	[23]
G**S**_53_T	*T. gammatolerans*	TgAmut	60	6.8	962	[23]
D**S**_55_T	*T. sibiricus*	TsAwt	90	3.0	2066.1	[16], this study
G**S**_55_Q	*T. sibiricus*	TsAmut	90	6.0	5037.7	This study

* G**S**Q—mesophilic, D**S**T—thermophilic, other combinations are “mixed”.

## Data Availability

The data presented in this study are contained within the article.

## Supplementary Materials

The supporting information can be downloaded at: https://www.mdpi.com/article/10.3390/ijms24119632/s1.
