# Supplementary material for: Enhancing the Catalytic Activity of Thermo-Asparaginase from Thermococcus sibiricus by a Double Mesophilic-like Mutation in the Substrate-Binding Region
Source: Int J Mol Sci. 2023 Jun 1;24(11):9632. doi: 10.3390/ijms24119632 (PMC10253665; doi:10.3390/ijms24119632)
Supplement: Supplementary file 1 [file ijms-24-09632-s001.zip › Supplementary Materials Figure S1.pdf]

Figure S1. Amino acid sequence comparison of mesophilic and thermophilic L-asparaginases

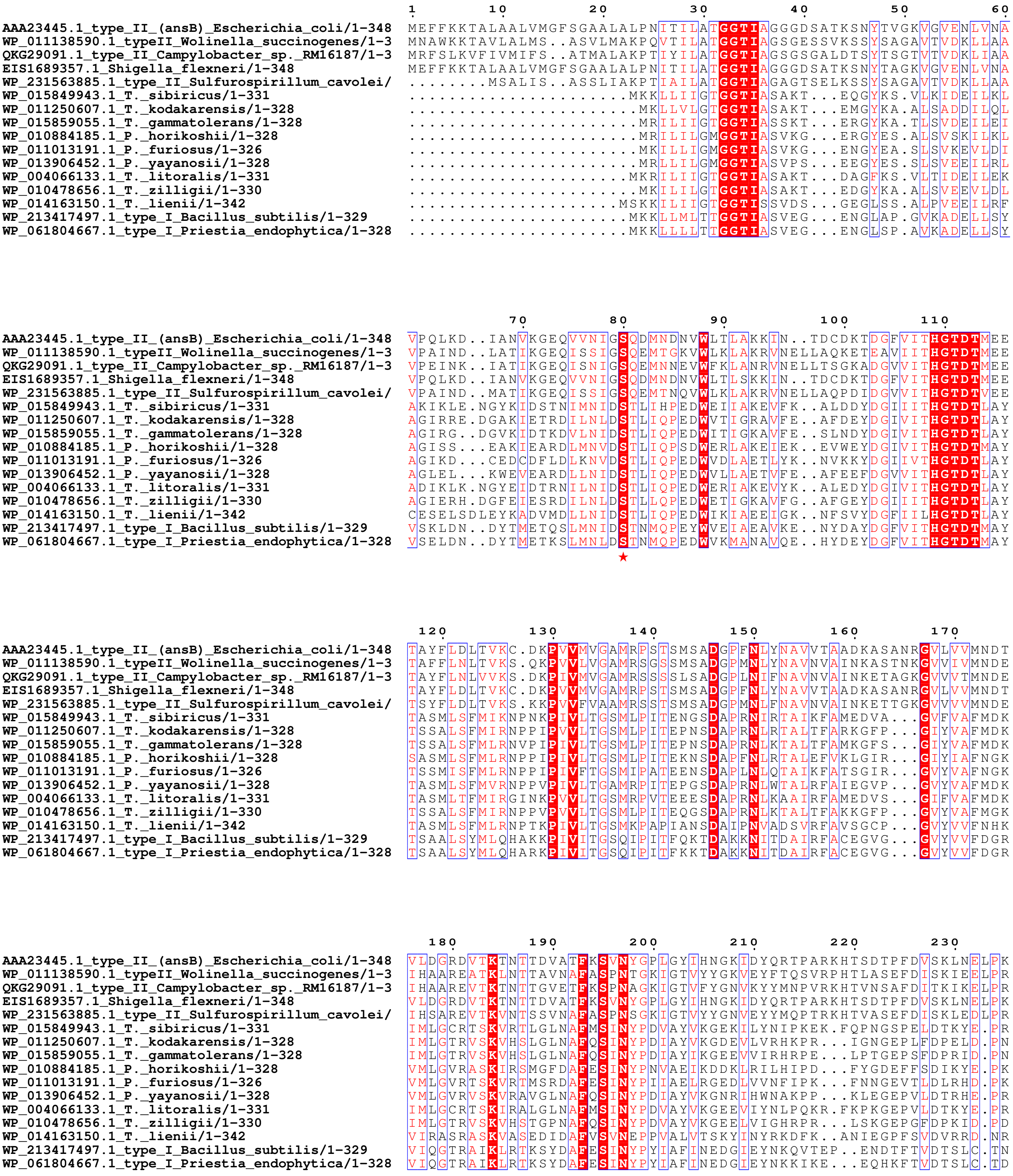

```
                                                     240       250       260       270       280       290
AAA23445.1_type_II_(ansB)_Escherichia_coli/1-348     VGIVYNYANASDLPAKALVDAGYDGIVSAGVGNGNL...YKSVFDTLATAAKTGTAVVRS
WP_011138590.1_typeII_Wolinella_succinogenes/1-3     VDILYAHPDDTDVLVNAALQAGAKGIIHAGMGNGNP...FPLTQNALEKAAKSGVVVARS
QKG29091.1_type_II_Campylobacter_sp._RM16187/1-3     VDIIYSHSNDNPDFANISIKNGAKGIISAGMGNGNP...FPTVLEVLGEGVKKGVVVVRD
EIS1689357.1_Shigella_flexneri/1-348                 VGIVYNYANASDLPAKALVDAGYDGIVSAGVGNGNL...YKSVFDTLATAAKTGTAVVRS
WP_231563885.1_type_II_Sulfurospirillum_cavolei/     VDIVYAHPSDTDVMVKAAVAAGAKGIIHAGMGNGNP...FPLTQEALAEAVKKGVIVARS
WP_015849943.1_T._sibiricus/1-331                    VVVLRVTPGLGGEIIDAVLDAGYKGIVLEGYGAGGLPYRKSNLLSKIKEITPK.IPVIMT
WP_011250607.1_T._kodakarensis/1-328                 VVHIRLTPGLSPEVLRAVARA.TDGIVLEGYGAGGIPYRGRNLLEVVSETARE.KPVVMT
WP_015859055.1_T._gammatolerans/1-328                VAYLRLTPGLSPEVFLAVAEK.VHGIVLEGYGAGGIPYRGRNLLEAVSRVARE.KPVVMT
WP_010884185.1_P._horikoshii/1-328                   VLVIKLIPGLSGDIVREALRLGYKGIILEGYGVGGIPYRGTDLFEVVSSISKR.IPVVLT
WP_011013191.1_P._furiosus/1-326                     VLVIKLIPGLSGDIFRAAVELGYRGIVIEGYGAGGIPYRGSDLLQTIEELSKE.IPIVMT
WP_013906452.1_P._yayanosii/1-328                    VLVLRLVPGMEGDVLEAALELGYRGIVLEGYGVGGIPYRGRDLLDVVRRVATE.IPVVMT
WP_004066133.1_T._litoralis/1-331                    VVFIRLTPGLDGNAIDALVKAGYRGIVLEGYGAGGIPYRKRDILSKIREITPQ.IPVVMT
WP_010478656.1_T._zilligii/1-330                     VAYLRLTPGLSPEVFLAVANK.AHGIILEGYGAGGIPYRGRDLLSAVSEVARE.KPVVMT
WP_014163150.1_T._lienii/1-342                       VFVLKIFPGLEPSIVETVIERDISGLIVESFGAGGLPYRGRNLLDILSKAAAQ.IPVVLT
WP_213417497.1_type_I_Bacillus_subtilis/1-329        VCLLKLHPGLKPEMFDALKSM.YKGIVIESYGSGGVPFEGRDILSKVNELIESGIVVVIT
WP_061804667.1_type_I_Priestia_endophytica/1-328     VLLLRLHPGLKPEMFDALKGL.YKGIVIESYGSGGIPFEERNILEKINELVESGVVVVIT
```

```
                                                           300       310       320       330       340
AAA23445.1_type_II_(ansB)_Escherichia_coli/1-348     SRVPTGA...TTQDAEVDDAKYGFVASGTLNPQKARVLLQLALTQTKDPQQIQQIFNQY.
WP_011138590.1_typeII_Wolinella_succinogenes/1-3     SRVGSGS...TTQEAEVDDKKLGFVATESLNPQKARVLLMLALTKTSDREAIQKIFSTY.
QKG29091.1_type_II_Campylobacter_sp._RM16187/1-3     SRVGSGE...TTLNGEVDDGKYGFLASDNLNAQKARVLLMLALTQTNDKAKIQEFFYTH.
EIS1689357.1_Shigella_flexneri/1-348                 SRVPTGA...TTQDAEVDDAKYGFVASGTLNPQKARVLLQLALTQTKDPQQIQQIFNQY.
WP_231563885.1_type_II_Sulfurospirillum_cavolei/     SRVGSGS...TTLEGEVDDAKYGFIATTTLNPQKARVLLMLGLTKTNDKKALQKLFLEY.
WP_015849943.1_T._sibiricus/1-331                    TQALYDGVDMRKYEVGRKALETGIIPAKDMTKEATITKLMWALGHTKDVEKIREIMHTNY
WP_011250607.1_T._kodakarensis/1-328                 TQALYGGVDLTRYEVGRRALEAGVIPAGDMTKEATLTKLMWALGHTRDLEEIRKIMERNI
WP_015859055.1_T._gammatolerans/1-328                TQALYGGVDLRRYEVGRRALEAGVIPAGDMTKEATLVKLMYALGRTNGLEEVRRIMEKNL
WP_010884185.1_P._horikoshii/1-328                   TQAIYDGVDLQRYKVGRIALEAGVIPAGDMTKEATITKLMWILGHTKNIEEVKQLMGKNI
WP_011013191.1_P._furiosus/1-326                     TQAMYDGVDLTRYKVGRLALRAGVIPAGDMTKEATVTKLMWILGHTNNVEEIKVLMRKNL
WP_013906452.1_P._yayanosii/1-328                    TQTLYDGVDLTKYKVGRKALEVGVIPAGDMTKEATITKLMWILGHTRDVGEVRRLMLTNM
WP_004066133.1_T._litoralis/1-331                    TQALYDGVDLTKYEVGRKALEAGIIPAKDMTKETTITKLMWALGHTKNVEEVKEIMHTNY
WP_010478656.1_T._zilligii/1-330                     TQALHGGVDLMKYEVGRRALEAGVIPAGDMTKEATLVKLMWALGHTKNVDEVREIILTNV
WP_014163150.1_T._lienii/1-342                       SQVLYNGVNLHTYEVGIRALKAGVISAEDMSKEAAVTKLMWVLGHTKNIDEIRRLFKTPI
WP_213417497.1_type_I_Bacillus_subtilis/1-329        TQCLEEGEDMSIYEVGRRVNQDLIIRSRNMNTEAIVPKLMWALGQSSDLPVVKRIMETPI
WP_061804667.1_type_I_Priestia_endophytica/1-328     TQCLEEGEDMSIYEVGRRVNQDLIIRSRNMNTEAIVPKLMWALGQSPDLHEVKRIMETPV
```

```
AAA23445.1_type_II_(ansB)_Escherichia_coli/1-348     ................
WP_011138590.1_typeII_Wolinella_succinogenes/1-3     ................
QKG29091.1_type_II_Campylobacter_sp._RM16187/1-3     ................
EIS1689357.1_Shigella_flexneri/1-348                 ................
WP_231563885.1_type_II_Sulfurospirillum_cavolei/     ................
WP_015849943.1_T._sibiricus/1-331                    VNEIKS..........
WP_011250607.1_T._kodakarensis/1-328                 AGEITGS.........
WP_015859055.1_T._gammatolerans/1-328                AGELSSAF........
WP_010884185.1_P._horikoshii/1-328                   TGELTRVS........
WP_011013191.1_P._furiosus/1-326                     VGELRD..........
WP_013906452.1_P._yayanosii/1-328                    VGEIGKSA........
WP_004066133.1_T._litoralis/1-331                    ADEIEG..........
WP_010478656.1_T._zilligii/1-330                     SDEIGKKES.......
WP_014163150.1_T._lienii/1-342                       AGEIKQHKIYYRTPQR
WP_213417497.1_type_I_Bacillus_subtilis/1-329        ADDVVL..........
WP_061804667.1_type_I_Priestia_endophytica/1-328     ADDVVL..........
```

Identical amino acid residues are marked in red. The substrate-binding Ser residue is marked by a red star. ESPript 3.0 was used for multiple sequence alignments [28].
