# Supplementary figures and images for: Enhancing the Catalytic Activity of Thermo-Asparaginase from Thermococcus sibiricus by a Double Mesophilic-like Mutation in the Substrate-Binding Region
Source: Int J Mol Sci. 2023 Jun 1;24(11):9632. doi: 10.3390/ijms24119632 (PMC10253665; doi:10.3390/ijms24119632)

**Figure S2.** Cytotoxic activity of TsA-D54G/T56Q against cancer cell lines.

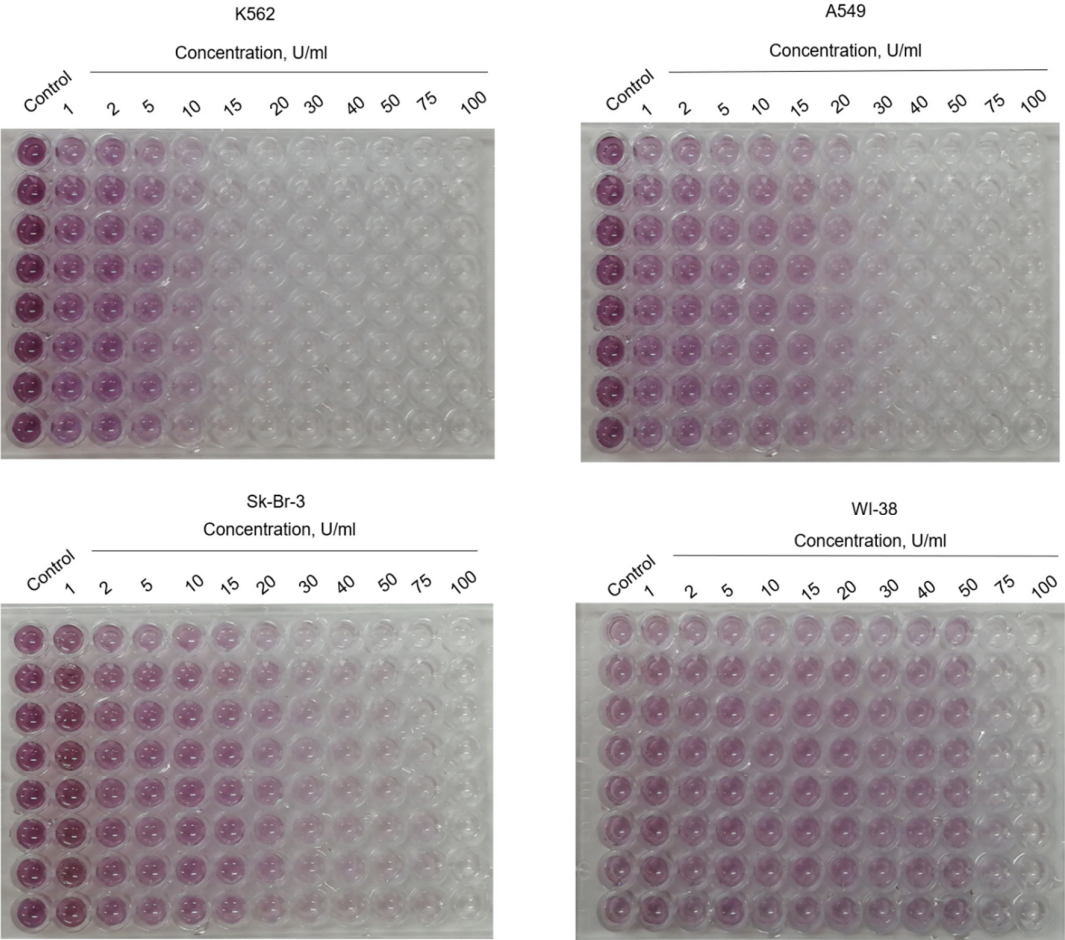

Supplement: Supplementary file 1 [file ijms-24-09632-s001.zip › Supplementary Materials Figure S2.pdf]
